# Influence of Genetic Variants of the N-Methyl-D-Aspartate Receptor on Emotion and Social Behavior in Adolescents

**DOI:** 10.1155/2016/6851592

**Published:** 2015-12-24

**Authors:** Li-Ching Lee, Ying-Chun Cho, Pei-Jung Lin, Ting-Chi Yeh, Chun-Yen Chang, Ting-Kuang Yeh

**Affiliations:** ^1^Science Education Center and Graduate Institute of Science Education, National Taiwan Normal University, 88 Section 4, Ting-Chou Road, Taipei 116, Taiwan; ^2^Graduate Institute of Clinical Medicine, College of Medicine, National Taiwan University Hospital, No. 1, Section 1, Jen-Ai Road, Taipei 100, Taiwan; ^3^Department of Pediatrics, Mackay Memorial Hospital, 92 Section 2, Zhongshan N. Road, Taipei 104, Taiwan; ^4^Department of Earth Sciences, National Taiwan Normal University, 88 Section 4, Ting-Chou Road, Taipei 116, Taiwan; ^5^Institute of Marine Environmental Science and Technology, National Taiwan Normal University, 88 Section 4, Ting-Chou Road, Taipei 116, Taiwan

## Abstract

Considerable evidence has suggested that the epigenetic regulation of N-methyl-D-aspartate (NMDA) glutamate receptors plays a crucial role in neuropsychiatric disorders. Previous exploratory studies have been primarily based on evidence from patients and have rarely sampled the general population. This exploratory study examined the relationship of single-nucleotide polymorphism (SNP) variations in the genes encoding the NMDA receptor (i.e., *GRIN1, GRIN2A, GRIN2B, GRIN2C*, and *GRIN2D*) with emotion and social behavior in adolescents. For this study, 832 tenth-grade Taiwanese volunteers were recruited, and their scores from the Beck Youth Inventories were used to evaluate their emotional and social impairments. Based on these scores, *GRIN1* (rs4880213) was significantly associated with depression and disruptive behavior. In addition, *GRIN2B* (rs7301328) was significantly associated with disruptive behavior. Because emotional and social impairment greatly influence learning ability, the findings of this study provide important information for clinical treatment and the development of promising prevention and treatment strategies, especially in the area of psychological adjustment.

## 1. Introduction

Interest in the pathology of emotional disorders, such as depression and anxiety, has increased, primarily because the incidence of emotional disorders in adults, adolescents, and even children has dramatically increased over the past several decades [1]. Emotional disorders are often influenced by genetic and lifestyle factors [2, 3], and understanding the genetic etiologies of these diseases could provide valuable information for the development of effective therapies.

The neuronal N-methyl-D-aspartate receptor (NMDAR) has been postulated to play a key role in the pathophysiology of schizophrenia, bipolar disorder, and depression [4, 5]. The possible role of NMDAR signaling in the pathophysiology of emotional disorders has been supported by the following evidence: (a) bipolar disorder and major depression disorder are associated with altered levels of central excitation neurotransmitters [6, 7], (b) NMDAR expression, distribution, and function are decreased in patients with mood disorders [8], (c) the NMDAR modulator exerts a positive therapeutic effect on patients [9], and (d) antidepressants and mood stabilizers can improve NMDAR function [10, 11]. Therefore, genes involved in the NMDAR pathway might be important genetic regulators of human physiology that consequently influence mood diseases.

**Figure 1:**
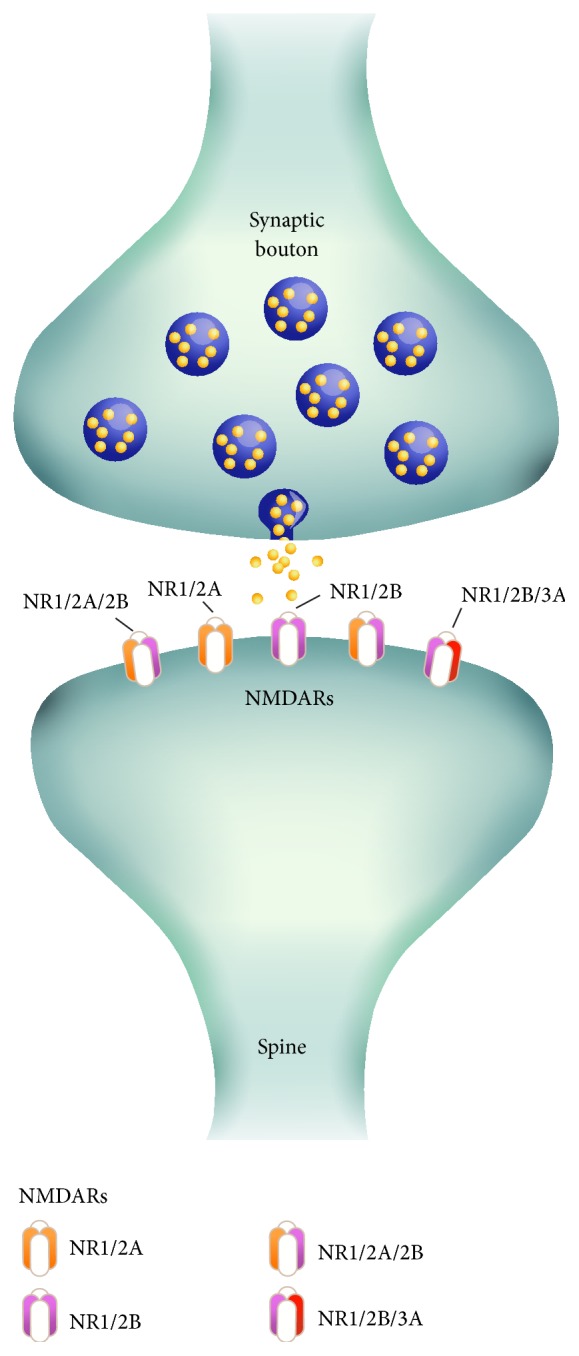
Different NMDA receptor stoichiometric subunits.

Recent studies have shown that many of the physiological and pharmacological properties of the NMDAR depend on the composition of its subunits [12]. NMDAR subtypes include at least one obligatory NR1 subunit in combination with different constellations of NR2 (i.e., GRIN2A, GRIN2B, GRIN2C, and GRIN2D) [13–15] and/or NR3 (i.e., GRIN3A and 3B) subunits. The alternative splicing of the* GRIN1* gene yields eight different NR1 isoforms that form different combinations with NR2 and/or NR3. These isoforms display unique properties in the central nervous system that depend on their subunit composition (Figure 1). The NR1 subunit appears to be a common prerequisite for the expression of functional NMDARs, and the NR2 subunit is required for the efficient formation of functional NMDARs. Moreover, the NR2 subunits determine biophysiological ion channel properties, such as the mean conductance open time and sensitivity to an Mg^2+^ block [16]. The NR2 subunit assembly process may also be a critical factor in postsynaptic signaling pathways that direct synaptic plasticity [17]. The dysfunction of NMDA ligand-gated cation channels is an underling molecular mechanism for neurologic disorders, such as schizophrenia [14], psychiatric disorders [18], and neurodegenerative diseases [19]. Recent studies indicated that alterations in NR1 and NR2 transcript expression associated with the NMDAR stoichiometry in schizophrenia involved more complex cellular changes than previously assumed [20, 21]. The heteromeric nature of NMDARs provides a wide variety of receptor subtypes; thus, single-nucleotide polymorphisms (SNPs) in NMDAR subunits are likely responsible for creating various distinct NMDAR properties. Several SNPs in the NMDAR subunit genes have been shown to affect the role of NMDAR signaling in the pathophysiology of mood disorders by altering the expression, distribution, and function of the NMDARs [4, 22].

Two SNPs of* GRIN1* merit special attention: rs11146020 (G1001C) and rs4880213 (*GRIN1* 5′-upstream region). The* GRIN1* (rs11146020) gene is located at 9q34, a locus in the promoter region of the NMDAR subunit that has been linked to schizophrenia [23, 24]. The* GRIN1* (rs11146020) gene product plays a fundamental role in many brain functions, and its involvement in the pathogenesis of schizophrenia has been widely investigated [25–27]. Only a few studies have focused on* GRIN1* rs4880213 and the pathogenesis of schizophrenia or mood disorders. Two studies showed that individuals with the* GRIN1* rs4880213 C/C variation displayed more severe disability and reduced NMDAR-mediated cortical excitability than individuals with the C/T or T/T variation [28, 29].

Further studies revealed that variations in the NMDAR 2B subunit gene (*GRIN2B*) were associated with schizophrenia, psychiatric disorders, and brain plasticity. For example, in human attention performance studies, subjects that were homozygous for the frequent C allele of the* GRIN2B* rs1806201 variation displayed more altered network scores than subjects in the other two genotype groups (C/T and T/T) [30]. The other 2B subunits of NMDAR have promising candidate SNP variants (i.e., rs1805476, rs1805477, rs1805501, and rs1805502) that affect the genetic susceptibility to obsessive-compulsive disorder [31]. In a functional study of protein expression, the* GRIN2B* rs1805502 (T5988C) C allele was associated with reduced* GRIN1* mRNA and protein expression in schizophrenic patients [5]. In pharmacological interventions, the same variant of NMDAR (*GRIN2B*) has been proposed to act as a genetic predictor of treatment-resistant depression (TRD) in patients with major depressive disorder (MDD) [32].

Understanding the involvement of* GRIN* genes in neurocognitive deficits will shed light on the importance of links between subunit involvement in plasticity paradigms and behavior. However, most previous studies of SNP associations with schizophrenia only investigated one or two genes in NMDARs, particularly* GRIN1*,* GRIN2A*, and* GRIN2B* [5, 24, 33]. Therefore, in this study, we used more extensive systemic sequencing to identify SNPs that may affect multiple genes related to NMDARs. Our results further elucidate the role of NMDAR signaling in emotion and social behavior.

This exploratory study examined the relationship of SNP variations in NMDAR genes with emotion and social behavior in adolescents. Data from randomized controlled trials could provide convincing evidence on the mechanisms of genetic and epigenetic effects on emotion [34]. However, previous exploratory studies have been primarily based on evidence from patients and have rarely sampled the general population. In addition, previous studies of schizophrenia revealed that the total number of NMDARs remains normal as the patient ages, but the receptor stoichiometry appears to change with age [35]. Therefore, an investigation of the impact of epigenetic changes on emotion and behavior in different cross sections of the population could be important for understanding the underlying mechanisms of mood disorders. To the best of our knowledge, the association between genetic SNPs and emotion in adolescents has not yet been investigated. This study aimed to fill this gap. Furthermore, epidemiological studies have linked mood disorders to gender differences. Specifically, women are more likely than men to be diagnosed with depression [36, 37]. Therefore, the current study aimed to control for the influence of gender on the effect of genetic polymorphisms on emotion using statistics.

## 2. Materials and Methods

### 2.1. Participants

Three public senior high schools were selected (one in southern Taiwan, one in central Taiwan, and one in northern Taiwan). A total of 832 tenth-grade volunteers, 269 of whom were male, were recruited for this study. The mean age of the subjects was 16.3 years (SD: 0.5; age range: 16-17 years). The volunteers and their parents were explicitly informed about the plan, protocol, and procedure for the study, and written consent was obtained prior to the start of the study. This study was approved by the institutional review board of the National Taiwan University Hospital and the ClinicalTrials.gov registry of clinical trials (ClinicalTrials.gov identifier: NCT00713570).

### 2.2. Gene Screening, Variation Analysis, and Bioinformatics

We investigated the association of 59 SNPs in the NMDAR genes (i.e.,* GRIN1*,* GRIN2A*,* GRIN2B*,* GRIN2C*, and* GRIN2D*) with emotion and behavior in 832 Han Chinese subjects. Fifty-nine SNPs from the entire set of candidate genes associated with NMDARs were identified based on information available in the Entrez Gene (http://www.ncbi.nlm.nih.gov/gene), HapMap (http://www.hapmap.org), and Ensembl (http://www.ensembl.org/Homo_sapiens) databases. In a pilot study, we analyzed genotypes by sequencing DNA from 20 subjects, and we assumed that some genetic polymorphisms involved in functional NMDAR subunits might contribute to individual differences in emotion and social behavior (GSJUNIOR_A16, Roche Union ClinBio Co. Ltd.). SNPs with a minor allele frequency greater than 5% in the pilot study that were identified in the analyzed samples were considered the most promising candidates, and representative common variants were selected. From the pilot study, 8 SNPs from the* GRIN1* and* GRIN2* genes were identified as the most promising candidates. The samples were genotyped by DNA sequencing of the relevant PCR products using an ABI Prism_ BigDye Terminator v3.1 Cycle Sequencing Ready Reaction kit and an ABI Prism_3730 Genetic Analyzer (Applied Biosystems, Foster City, CA, USA). All participants were screened for the 8 selected SNPs using a custom/commercial TaqMan SNP genotyping assay according to the manufacturer's instructions (Life Technologies, Carlsbad, CA, USA) and a ViiA 7 real-time PCR system (Life Technologies).

### 2.3. Emotion Assessment

The subjects' emotional behaviors were assessed via the second edition of the complete Beck Youth Inventories (BYI-II) [38], which is a commonly used and thoroughly standardized test that suitably assesses the diversity of emotion and behavior in adolescents. The BYI-II consists of five inventories: anxiety, depression, anger, disruptive behavior, and self-concept, and each of these inventories contains 20 items that are assessed based on five self-reported scales. Higher scores for the inventories of anxiety, depression, anger, or disruptive behavior indicate a tendency towards emotional instability. In contrast, a higher score in the self-concept inventory reveals that the student has a positive sense of self.

### 2.4. Statistical Analysis

Two-tailed* t*-tests were used to assess the significance of differences in the BYI-II subtask scores between males and females. For each SNP, the participants were assigned to one of three groups based on their genotype, and deviation from the Hardy-Weinberg equilibrium was tested using a chi-squared test. An analysis of covariance (ANCOVA) was employed to determine the effect of each SNP genotype on behavior based on the BYI-II subtask scores. Previous studies revealed that gender is an important determinant of emotion. To assess the effect of genetic polymorphism on emotion, the gender of participants served as a covariate in this analysis to control for gender effects on BYI-II subtask scores. Post hoc analyses using pairwise comparisons were also used to identify trends in the data. The level of confidence was set at the 0.05 significance level. In consideration of type I errors, permutation tests (with *N* = 1,000 randomizations) [39] were performed to correct for multiple comparisons. The assumptions used for the ANCOVA and inferential statistical analyses were tested using SPSS version 22.0.

## 3. Results

NMDARs are tetramers that contain two GRIN1 subunits bound to two GRIN2 (A, B, C, or D) and/or GRIN3 (A or B) subunits. Many of the physiological and pharmacological properties of NMDARs depend on the specific GRIN2 and GRIN3 subunits. The observed distributions of the genotypes for each SNP appeared to follow Hardy-Weinberg equilibrium. The analyses of SNPs in NMDAR-related genes, genotypic distribution, chromosome localization, and genotype frequency are presented in Table 1.

The demographic and BYI-II subtask scores of the participants are outlined in Table 2. Some differences between males and females were observed. Female subjects in this study tended to be more anxious, whereas male subjects exhibited more disruptive behaviors.

As shown in Table 3, the emotional instability significantly differed in individuals carrying the* GRIN1* (rs4880213) and* GRIN2B* (rs7301328) polymorphisms. After the multiple comparison correction by 1,000 permutation tests,* GRIN1* (rs4880213) was significantly associated with depression (*P* = 0.04) and disruptive behavior (*P* = 0.04), and* GRIN2B* (rs7301328) was significantly associated with disruptive behavior (*P* = 0.05). The other six SNPs were not significantly associated with behavior based on the BYI-II analysis.

The ANCOVA of the depression subtask scores revealed a significant main effect for the three genotype groups of* GRIN1* (rs4880213; Table 4,* F*[2,829] = 4.5, *P* < 0.05). The pairwise test revealed that the depression subtask scores of the T/T genotype group were significantly lower than those of the C/C and C/T genotype groups (Table 5, C/C > C/T, *P* = 0.005; C/T > T/T, *P* = 0.04). The* GRIN1* (rs4880213) polymorphisms also influenced the disruptive behavior scores (Table 6,* F*[2,829] = 4.0, *P* < 0.05). Subjects with the T/T genotype group tended to score lower on the disruptive behavior subtask (Table 7, C/T > T/T, *P* = 0.005). Furthermore, the disruptive behavior scores were significantly associated with the* GRIN2B* rs7301328 polymorphism (Table 8,* F*[2,829] = 4.2, *P* = 0.05). As shown in Table 9, a pairwise comparison analysis revealed that the disruptive behavior subtask scores of the G/G genotype group were significantly lower than those of the C/C genotype group (*P* = 0.004).

## 4. Discussion

All NMDARs appear to function as heteromeric assemblies that consist of multiple NR1 subunits and at least one type of NR2. The NR3 subunit does not form functional receptors alone but can coassemble with NR1/NR2 complexes. The temporal and spatial expression patterns of the NR2 and NR3 subunits were recently shown to differ in the brain, and the expression of NMDAR subtypes also vary by cell type and subcellular localization [40, 41].* In situ* hybridization studies have shown that the mRNAs for NMDAR subunits are differentially distributed throughout the brain, and the expression patterns of these mRNAs change strikingly during development [42]. This study aimed to determine whether any other SNPs are responsible for the distinct properties of the NMDAR that are associated with emotion and behavior, especially in adolescents. Previous studies show that* GRIN1* (rs11146020) was strongly associated with bipolar disorder [4] and depressive symptoms [43]. The incidences of depression and disruptive behavior were significantly lower in participants that carried the* GRIN1* T/T genotype (SNP rs4880213) than in members of the two other groups; this result was consistent with Georgi's study, which showed that the T allele was less frequent in schizophrenia patients with a lifetime history of depression than in control. Francesco et al. [44] suggested that the T/T genotype of the* GRIN1* rs4880213 SNP is associated with reduced intracortical inhibition, enhanced glutamatergic excitation, and enhanced glutamate NMDAR function. Francesco's study also revealed that the* GRIN1* and* GRIN2B* subunits of NMDARs are involved in regulating cortical excitability and plasticity in the human cortex. Rossi et al. [28] also indicated that the C allele of rs4880213 is associated with reduced NMDAR-mediated cortical excitability. Notably, a number of studies have indicated that NMDAR dysfunction is associated with depression syndrome [10]. Thus, the homozygosity of the* GRIN1* rs4880213 T allele might increase glutamate NMDAR function and stabilize the mood status compared with other alleles. However, because the effect of the* GRIN1* rs4880213 SNP on NMDAR function has not yet been analyzed at the molecular or metalevel, the mechanism by which the* GRIN1* rs4880213 SNP affects NMDAR function needs to be investigated.

Our results also showed that the homozygosity of the C allele of the* GRIN2B* (rs7301328) is associated with increases in disruptive behavior; this finding is consistent with a report by Ohtsuki et al. [45], which showed that the G allele of the 366C/G (rs7301328) polymorphism is more common in patients than in population-based controls. The* GRIN2B* SNP (rs7301328) has been linked to bipolar disorder [24], schizophrenia, and other neuropsychiatric disorders; however, to the best of our knowledge, few studies have investigated the influence of the* GRIN2B* SNP (rs7301328) at the molecular level, including its effect on NMDAR function or on the level of* GRIN1* or* GRIN2* expression. In addition, the currently available studies do not conclusively show that unique* GRIN2* subunits selectively mediate directions of plasticity, especially regarding long-term depression (LTD). In some studies, behavioral impairment can be related to a selective deficit in one direction of plasticity. Moreover, a* GRIN2B*-dependent LTD-like process has also often been implicated in mechanisms that support reversal learning [4, 46]. A rigorous testing of this hypothesis based on an analysis of more complete information concerning the effect of the* GRIN2B* SNP (rs7301328) would be interesting.

This study did not identify significant associations between the other polymorphisms and behavioral indices. Previous exploratory studies have indicated that* GRIN1* (rs11146020) is a good candidate for susceptibility to schizophrenia [24, 25, 33], and other studies have suggested that the combined effects of* GRIN1* and* GRIN2B* gene polymorphisms, including rs1805247 and rs1805502, might be involved in the etiology of schizophrenia. However, we did not identify such an association. Notably, the students in this study were physically and psychologically healthy, and identifying significant differences in emotional performance among different genotype groups is more difficult in a healthy population than in patients who are suffering from single-gene diseases or psychiatric disorders.

Here, we report an exploratory study of SNPs in the NMDAR* GRIN1* and* GRIN2* subunit genes in a healthy adolescent population. Specifically, we verified that the two SNPs (rs4880213 and rs7301328) influenced emotional performance in this adolescent population. Nevertheless, this study is subject to some limitations. First, most of the participants were relatively physically healthy. Nevertheless, the effect size for the association between the examined SNPs and behavior was narrow. However, because observing significant differences within a normal population is difficult, future studies may rely on the data presented herein to explore mechanisms relevant to this association, both at the clinical and molecular levels. The other limitation of the present study is differences in the socioeconomic levels of students which may have influenced the emotional behavior assessments. All volunteers in this study lived in metropolitan areas of Taiwan. Studies showed students socioeconomic levels were likely approximately equivalent in metropolitan areas of Taiwan [47]. Therefore, the impact of social economy on emotion in this study might be less significant. However, because the current study did not measure the effects of socioeconomic status on emotional behavior, we cannot conclusively rule out such an effect. Subsequent studies should rigorously examine this effect.

Educational researchers, teachers, and counselors might be interested in the implications of the effects of genetic polymorphisms identified herein. Specifically, students who do not perform as well as others academically due to poor cognitive abilities or emotional self-control show a decreased willingness to learn. Genotyping these students could provide educators with a strategic understanding of the potential innate emotional self-control of an individual student. Because human emotion is influenced by interactions between genetic variations and environmental factors, this knowledge could be used to provide an appropriate environment and monitor the emotions of a student during learning. These strategies could improve a student's interest in learning and achievement.

## Supplementary Material

We investigated the association of 59 SNPs in the NMDAR genes (i.e., GRIN1, GRIN2A, GRIN2B, GRIN2C, and GRIN2D) with emotion and behavior in 832 Han Chinese subjects. Fifty-nine SNPs from the entire set of candidate genes associated with NMDARs were identified as shown supplementary material information in terms of their dbSNP IDs, genomic location, variation, and allele frequency.

## Figures and Tables

**Table 1:** The genotype distributions and chromosome locations of the SNPs.

Gene/SNP ID	Allele/genotype	Subjects	Chromosome region	Genotype frequency	CHB population frequency^*∗*^
*GRIN1 *			9q34.3		
rs4880213	CC/CT/TT	124/368/340		0.15/0.44/0.41	0.11/0.44/0.45
rs11146020	CC/CG/GG	565/239/28		0.68/0.29/0.03	0.72/0.24/0.04
*GRIN2B *			12p12		
rs7301328	CC/CG/GG	250/396/186		0.30/0.48/0.22	0.26/0.43/0.31
rs1806201	AA/AG/GG	223/438/171		0.27/0.53/0.20	0.22/0.55/0.22
rs1805247	AA/AG/GG	608/204/20		0.73/0.25/0.02	0.61/0.35/0.04
rs1805502	AA/AG/GG	608/203/20		0.73/0.24/0.03	0.61/0.35/0.04
rs3764028	AA/AC/CC	148/397/287		0.18/0.48/0.34	0.13/0.46/0.42
*GRIN2C *			17q25		
rs3744215	AA/AC/CC	143/423/266		0.17/0.51/0.32	0.15/0.47/0.38

^*∗*^Data from 1000 Genomes Project Phase 3; http://www.1000genomes.org/.

**Table 2:** Distribution of selected characteristics of the participants.

	Male	Female	*P*
Age (yrs)	16.8 (0.32)^*∗*^	16.8 (0.30)	
*BYI-II *			
Anxiety	16.2 (8.9)	18.5 (8.8)	<0.01
Depression	13.6 (9.4)	15.1 (9.3)	
Anger	11.7 (8.9)	12.4 (7.8)	
Disruptive behavior	8.2 (7.2)	6.6 (4.9)	<0.01
Self-concept	36.0 (9.6)	36.5 (7.9)	

^*∗*^Mean (SD).

**Table 3:** Genotype distributions and comparisons of subtask scores and genotype groupings.

	C/C	C/T	T/T	*F*	*P*_cor_
*GRIN1* rs4880213					
Anxiety	19.1 ± 9.5^*∗*^	18.2 ± 9.0	17.0 ± 8.5	2.6	>0.05
Depression	16.5 ± 9.8	15.0 ± 9.5	13.5 ± 8.8	4.6	**0.04**
Anger	12.3 ± 7.8	12.8 ± 8.3	11.6 ± 8.0	1.4	>0.05
Disruptive behavior	7.2 ± 5.6	7.7 ± 6.1	6.4 ± 5.1	4.1	**0.04**
Self-concept	36.0 ± 7.9	36.2 ± 9.2	36.7 ± 8.0	0.4	>0.05

	C/C	C/G	G/G		

*GRIN2B* rs7301328					
Anxiety	17.5 ± 8.6	17.8 ± 9.0	18.6 ± 9.3	0.8	>0.05
Depression	14.1 ± 8.9	14.6 ± 9.1	15.5 ± 10.5	1.1	>0.05
Anger	11.6 ± 7.8	12.3 ± 7.8	13.1 ± 9.2	1.5	>0.05
Disruptive behavior	6.3 ± 5.6	7.2 ± 5.6	7.9 ± 5.8	3.7	**0.05**
Self-concept	36.2 ± 8.9	36.9 ± 7.6	35.7 ± 9.5	1.2	>0.05

^*∗*^Mean values ± standard deviations.

*P*_cor_: multiple comparison correction by 1,000 permutation tests.

**Table 4:** ANCOVA summary for the depression subtask scores.

Source of variance	Df	SS	MS	*F*	*P*
*GRIN1* rs4880213	2	787.6	393.8	4.5	0.04^*∗*^
Gender	1	278.3	278.3	3.2	0.07
Residual	828	21794	26.3		

^*∗*^Multiple comparison correction by 1000 permutation tests.

**Table 5:** Pairwise comparisons of three* GRIN1* rs4880213 genotype groups in terms of adjustments of depression subtask scores.

*GRIN1* rs4880213	M_adj_	SE	Pairwise comparison
C/C	16.5	0.9	C/C > T/T (*P* = 0.005)
C/T	15.1	0.5	C/T > T/T (*P* = 0.04)
T/T	13.5	0.5	

**Table 6:** ANCOVA summary for the disruptive behavior subtask scores.

Source of variance	Df	SS	MS	*F*	*P*
*GRIN1* rs4880213	2	254.3	127.1	4.0	0.04^*∗*^
Gender	1	335.2	335.2	10.5	0.001
Residual	828	21794	26.5		

^*∗*^Multiple comparison correction by 1,000 permutation tests.

**Table 7:** Pairwise comparisons of three* GRIN1* rs4880213 genotype groups in terms of adjustments of disruptive behavior subtask scores.

*GRIN1* rs4880213	M_adj_	SE	Pairwise comparison
C/C	7.3	0.5	C/T > T/T (*P* = 0.005)
C/T	7.7	0.3	
T/T	6.4	0.3	

**Table 8:** ANCOVA summary for the disruptive behavior subtask scores.

Source of variance	Df	SS	MS	*F*	*P*
*GRIN2B* rs7301328	2	269.2	269.2	4.2	0.05^*∗*^
Gender	1	386.3	386.3	12.1	0.001
Residual	828	21779	26.3		

^*∗*^Multiple comparison correction by 1,000 permutation tests.

**Table 9:** Pairwise comparisons of three *GRIN2B rs7301328* genotype groups in terms of adjustments of disruptive behavior subtask scores.

*GRIN2B* rs7301328	M_adj_	SE	Pairwise comparison
C/C	6.2	0.5	C/C > G/G (*P* = 0.004)
C/G	7.2	0.3	
G/G	8.0	0.3	
