# Supplementary material for: Influence of Genetic Variants of the N-Methyl-D-Aspartate Receptor on Emotion and Social Behavior in Adolescents
Source: Neural Plast. 2015 Dec 24;2016:6851592. doi: 10.1155/2016/6851592 (PMC4706971; doi:10.1155/2016/6851592)
Supplement: Supplementary file 1 — We investigated the association of 59 SNPs in the NMDAR genes (i.e., GRIN1, GRIN2A, GRIN2B, GRIN2C, and GRIN2D) with emotion and behavior in 832 Han Chinese subjects. Fifty-nine SNPs from the entire set of candidate genes associated with NMDARs were identified as shown supplementary material information in terms of their dbSNP IDs, genomic location, variation, and allele frequency. [file 6851592.f1.docx]

**Supplement Table** 59 SNPs information in terms of their dbSNP IDs, genomic location, variation, and allele frequency

| Gene | position |  | location |  | SNP ID |  | Nucleotide position and allelic form |  | variation |  | Allele frequency |
| --- | --- | --- | --- | --- | --- | --- | --- | --- | --- | --- | --- |
| GRIN1 | chr9:137138632 | | 5'UTR |  | rs11146020 |  | -855 G/C |  |  |  | G:C=0.9:0.1 |
|  | chr9:137149070 | | Exon4 |  | rs11575901 |  | +632 C/T |  | 211 Ala>Val |  | C:T=1:0 |
|  | chr9:137149026 | | Exon4 |  | rs75484994 |  | +588 G/T |  | 196 Gln>His |  | G:T=1:0 |
|  | chr9:137156862 | | Intron5 |  | rs71508898 |  | +794-1 G/A |  | Splice Site |  | G:A=1:0 |
|  | chr9:137136549 | | 5' upstream |  | rs4880213 |  | C/T |  |  |  | C:T=0.2:0.8 |
|  |  |  |  |  |  |  |  |  |  |  |  |
| GRIN2A | chr16:10181870 | | Intron2 |  | rs11575875 |  | -19+7 G/T |  | Splice Site |  | G:T=1:0 |
|  | chr16:10180015 | | Exon3 |  | rs77288930 |  | +397 A/G |  | 133 Met>Val |  | A:G=1:0 |
|  | chr16:9938524 | | Exon4 |  | rs35898789 |  | +441_442 -/C |  | 148 Frameshift |  | -/C=1:0 |
|  | chr16:9891003 | | Exon5 |  | rs77772378 |  | +1105 G/A |  | 369 Asp>Asn |  | G:A=1:0 |
|  | chr16:9840943 | | Exon7 |  | rs112193824 |  | +1490 A/G |  | 497 Ile>Thr |  | A:G=1:0 |
|  | chr16:9834099 | | Intron9 |  | rs76549675 |  | +1777+6 G/T |  | Splice Site |  | G:T=1:0 |
|  | chr16:9822408 | | Exon11 |  | rs75130648 |  | +2024 A/C |  | 675 Asp>Ala |  | A:C=1:0 |
|  | chr16:9798282 | | Exon12 |  | rs76392464 |  | +2351 C/G |  | 784 Gly>Ala |  | C:G=1:0 |
|  | chr16:9768987 | | Exon13 |  | rs74458073 |  | +2459 A/C |  | 820 Val>Gly |  | A:C=1:0 |
|  | chr16:9764684 | | Exon14 |  | rs111354251 |  | +2860 T/C |  | 954 Ser>Gly |  | T:C=1:0 |
|  | chr16:9764645 | | Exon14 |  | rs61731465 |  | +2899 C/G |  | 967 Val>Leu |  | C:G=1:0 |
|  | chr16:9764316 | | Exon14 |  | rs61758995 |  | +3228 G/T |  | 1076 Asn>Lys |  | G:T=1:0 |
|  | chr16:9763966 | | Exon14 |  | rs75761674 |  | +3578 T/G |  | 1193 Leu>Trp |  | T:G=1:0 |
|  | chr16:9763771 | | Exon14 |  | rs56241810 |  | +3773 C/A |  | 1258 Gly>Val |  | C:A=1:0 |
|  | chr16:9763339 | | Exon14 |  | rs74935155 |  | +4205 G/A |  | 1402 Arg>Gln |  | G:A=1:0 |
|  | chr16:9763237 | | Exon14 |  | rs77029288 |  | +4307 A/G |  | 1436 Asn>Ser |  | A:G=1:0 |
|  |  |  |  |  |  |  |  |  |  |  |  |
| GRIN2B | chr12:13563041 | | Exon13 |  | rs1805247 |  | +4197 T/C |  | 1399 His (synonymous) |  | T:C=0.925:0.075 |
|  | chr12:13982130 | | 5'upstream |  | rs3764028 |  | C/A |  |  |  | A:C=0.325:0.675 |
|  | chr12:13561247 | | 3'UTR |  | rs1805502 |  | 1536 T/C |  |  |  | T:C=0.9:0.1 |
|  | chr12:13866148 | | Exon2 |  | rs79046967 |  | +61 G/A |  | 21 Val>Met |  | G:A=1:0 |
|  | chr12:13865843 | | Exon2 |  | rs7301328 |  | +366 C/G |  | 122 Pro (synonymous) | | C:G=0.525:0.475 |
|  | chr12:13753531 | | Exon3 |  | rs74987973 |  | +796 A/G |  | 266 Thr>Ala |  | A:G=1:0 |
|  | chr12:13753476 | | Exon3 |  | rs76180400 |  | +851 A/C |  | 284 Glu>Ala |  | A:C=1:0 |
|  | chr12:13615554 | | Exon6 |  | rs112992494 |  | +1439 A/G |  | 480 Leu>Pro |  | A:G=1:0 |
|  | chr12:13611779 | | Exon8 |  | rs118084307 |  | +1726 C/A |  | 576 Val>Phe |  | C:A=1:0 |
|  | chr12:13608793 | | Exon9 |  | rs35464354 |  | +1819 -/A |  | 607 Frameshift |  | -/A=1:0 |
|  | chr12:13567234 | | Exon12 |  | rs79163356 |  | +2389 C/T |  | 797 Leu>Phe |  | C:T=1:0 |
|  | chr12:13564574 | | Exon13 |  | rs1806201 |  | +2664 C/T |  | 888 Thr (synonymous) | | T:C=0.525:0.475 |
|  | chr12:13563555 | | Exon13 |  | rs75670883 |  | +3683 C/T |  | 1228 Thr>Met |  | C:T=1:0 |
|  | chr12:13562951 | | Exon13 |  | rs35260946 |  | +4286 -/G |  | 1429 Frameshift |  | -/G=1:0 |
|  |  |  |  |  |  |  |  |  |  |  |  |
| GRIN2C | chr17:74855071 | | Exon2 |  | rs80016270 |  | +22 G/A |  | 8 Ala>Thr |  | G:A=1:0 |
|  | chr17:74851653 | | Exon4 |  | rs77993162 |  | +1037 C/T |  | 346 Ser>Phe |  | C:T=1:0 |
|  | chr17:74850581 | | Exon5 |  | rs78647801 |  | +1300 C/T |  | 434 Arg>Cys |  | C:T=1:0 |
|  | chr17:74850361 | | Exon6 |  | rs61754645 |  | +1336 G/A |  | 446 Val>Met |  | C:T=1:0 |
|  | chr17:74850229 | | Exon6 |  | rs11575879 |  | +1468 G/A |  | 490 Val>Ile |  | G:A=1:0 |
|  | chr17:74846089 | | Exon11 |  | rs61740921 |  | +2327 G/A |  | 776 Ala>Val |  | G:A=1:0 |
|  | chr17:74842510 | | Exon13 |  | rs3744215 |  | +3627 G/T |  | 1209 Arg>Ser |  | G:T=0.512 : 0.488 |
|  | chr17:74842599 | | Exon13 |  | rs115230539 |  | +3538 C/T |  | 1180 Gly>Arg |  | C:T=1:0 |
|  |  |  |  |  |  |  |  |  |  |  |  |
| GRIN2D | chr19:48404826 | | Exon3 |  | rs35658494 |  | +558 -/C |  | 187 Frameshift |  | -/C=1:0 |
|  | chr19:48404830 | | Exon3 |  | rs77520296 |  | +562 G/T |  | 188 Val>Leu |  | G:T=1:0 |
|  | chr19:48404896 | | Exon3 |  | rs77698773 |  | +628 G/A |  | 210 Gly>Ser |  | G:A=1:0 |
|  | chr19:48414890 | | Exon6 |  | rs80005942 |  | +1439 A/T |  | 480 Glu>Val |  | A:T=1:0 |
|  | chr19:48414895 | | Exon6 |  | rs74672030 |  | +1444 C/T |  | 482 Arg>Cys |  | C:T=1:0 |
|  | chr19:48414964 | | Exon6 |  | rs77806672 |  | +1513 C/T |  | 505 Leu>Phe |  | C:T=1:0 |
|  | chr19:48419236 | | Exon8 |  | rs76227163 |  | +1738 C/T |  | 580 Pro>Ser |  | C:T=1:0 |
|  | chr19:48419256 | | Exon8 |  | rs78900630 |  | +1758 G/A |  | 586 Trp>*stopt gained | | G:A=1:0 |
| GRIN3A | chr9:101670974 | | Exon3 |  | rs149729514 |  | 1438 C/G |  | 480 Arg>Gly |  | C:G=1:0 |
|  | chr9:101623429 | | Exon5 |  | rs10989563 |  | 2503G/A |  | 835 Asp>Asn |  | G:A=0.958:0.041 |
|  |  |  |  |  |  |  |  |  |  |  |  |
| GRIN3B | chr19:1003163 | | Exon2 |  | rs113181909 |  | 460 C/T |  | 154 Pro>Ser |  | C:T=1:0 |
|  | chr19:1004724 | | Exon3 |  | rs144402697 |  | 1223 C/A |  | 408 Pro>Gln |  | C:A=1:0 |
|  | chr19:1007677 | | Exon4 |  | rs201299606 |  | 2102 G/T |  | 701 Ser>ILe |  | G:T=1:0 |
|  | chr19:1008217 | | Exon6 |  | rs139755201 |  | 2392 G/A |  | 798 Gly>Ser |  | G:A=1:0 |
|  | chr19:1008646 | | Exon7 |  | rs78914045 |  | 2495 C/A |  | 832 Ala>Glu |  | C:A=1:0 |
|  | chr19:1009204 | | Exon9 |  | rs200925289 |  | 2734 C/G |  | 912 Gln>Glu |  | C:G=1:0 |
